# Early neurological improvement with deferoxamine after intracerebral hemorrhage: A post hoc analysis of the i-DEF trial

**DOI:** 10.1177/17474930251348088

**Published:** 2025-05-26

**Authors:** Alexandros A Polymeris, Vasileios-Arsenios Lioutas, Lydia D Foster, Diego Incontri, Elizabeth C Heistand, Juliette Marchal, Alexa Lazar, Urs Fischer, Stefan T Engelter, David J Seiffge, Sharon D Yeatts, Magdy H Selim

**Affiliations:** 1 Stroke Division, Department of Neurology, Beth Israel Deaconess Medical Center, Harvard Medical School, Boston, MA, USA; 2 Department of Neurology and Stroke Center, University Hospital Basel and University of Basel, Basel, Switzerland; 3 Department of Public Health Sciences, Medical University of South Carolina, Charleston, SC, USA; 4 Department of Neurology, Inselspital, Bern University Hospital and University of Bern, Bern, Switzerland; 5 Department of Rehabilitation and Neurology, University Department of Geriatric Medicine FELIX PLATTER, University of Basel, Switzerland

**Keywords:** Intracerebral hemorrhage, early improvement, deferoxamine, trial

## Abstract

**Background:**

Little is known about early major neurological improvement (EMNI) after intracerebral hemorrhage (ICH).

**Aims:**

We performed a post hoc analysis of the Intracerebral Hemorrhage-Deferoxamine trial (i-DEF; NCT02175225) to comprehensively evaluate EMNI and assess whether deferoxamine treatment affects it.

**Methods:**

Comparing repeated assessments of National Institutes of Health Stroke Scale (NIHSS) on days 2, 3, 4, and 7 (or discharge, if it was earlier) versus NIHSS score at presentation, and defining EMNI as an NIHSS score decrement of an absolute ⩾4 points from presentation, we determined its presence or absence on day 2, day 3, day 4, and day 7(/discharge). Using adjusted generalized linear mixed-effects or logistic models as appropriate, we examined the association of deferoxamine with EMNI as repeated measure, as well as EMNI’s overall frequency, time course, determinants, and association with favorable long-term outcome (modified Rankin Scale 0–2).

**Results:**

Among 291 i-DEF participants in the modified intention-to-treat population (median age 61 years, 38.5% female, median NIHSS score 13, 144 randomized to deferoxamine and 147 to placebo), the proportion of participants with EMNI continuously increased from 20% on day 2 to 36% on day 7(/discharge). Deferoxamine was associated with an average twofold higher odds of EMNI (odds ratio (OR): 2.30, 95% confidence interval (CI): 1.07 to 4.95, p = 0.033 after adjustment for the prespecified trial covariates onset-to-treatment time, baseline ICH volume, and presenting NIHSS score), without clear evidence for treatment-by-time interaction (p_interaction_ = 0.092). Secondary and sensitivity analyses using alternative EMNI definitions (as relative ⩾20% or ⩾30% NIHSS score decrement) and additional covariate adjustment yielded consistent findings. Race, ICH volume and location were also associated with EMNI. EMNI was independently associated with twofold to sixfold higher odds of favorable 90-day and 180-day outcome, regardless of assessment timepoint.

**Conclusion:**

In a post hoc analysis of the i-DEF trial, the likelihood of EMNI over the first week following ICH was higher with deferoxamine. EMNI showed a continuous upward trajectory and strong association with favorable long-term functional outcome.

## Graphical abstract

**Graphical abstract:**
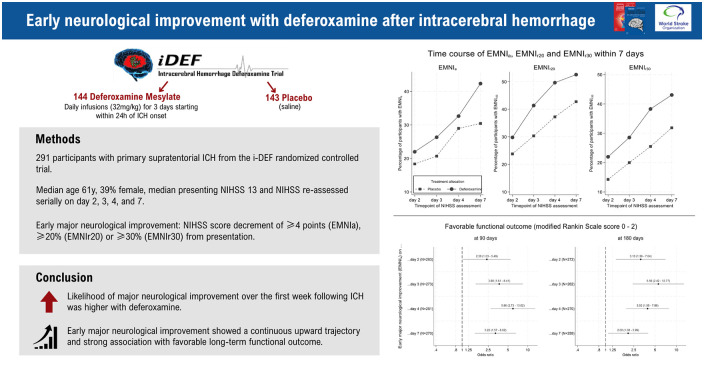

## Introduction

Intracerebral hemorrhage (ICH) is a devastating stroke form with sparse specific treatment options.^1^ The early – acute and subacute– phase following ICH is known to be highly dynamic, but research on early clinical changes after ICH has almost exclusively focused on neurological deterioration.^2–4^ However, many patients with ICH do not worsen and may even improve early on.^5,6^ Early major neurological improvement (EMNI) has been extensively studied in ischemic stroke, particularly as an outcome predictor and treatment outcome per se.^7,8^ Little is known about EMNI after ICH.^9^ Hardly any data exist about the time course and determinants of EMNI within the first week of ICH, its association with long-term outcomes, and how EMNI might be affected by treatments targeting secondary injury.^10^

Iron toxicity has been implicated in secondary brain injury after ICH.^11^ The Intracerebral Hemorrhage-Deferoxamine (i-DEF) randomized controlled trial showed signals for a positive effect of an early 3-day course of deferoxamine mesylate, an iron chelator, on functional outcomes and the trajectory of long-term recovery.^12,13^ In i-DEF, a substantially higher proportion of deferoxamine-treated participants achieved a modified Rankin Scale (mRS) score of 0–2, compared to placebo counterparts, within the first 7 days after ICH, indicating that deferoxamine might facilitate EMNI.^13^

## Aims

In this post hoc analysis of serial neurological assessments obtained using the National Institutes of Health Stroke Scale (NIHSS) over the first week of standardized follow-up within the i-DEF trial, we examined (1) the association of deferoxamine treatment with EMNI and (2) EMNI’s overall frequency, time course, determinants, and association with long-term outcome up to 180 days.

## Methods

### Study design and participants

Post hoc analysis of the i-DEF placebo-controlled, randomized, phase 2 clinical trial (NCT02175225) conducted across 40 centers in the United States and Canada. The trial methodology, data collection methods, and main results are described elsewhere.^12,14^ In short, i-DEF randomized participants aged 18–80 years with primary supratentorial ICH and without indication that withdrawal of care would be implemented within 72 h to receive daily intravenous infusions of deferoxamine (32 mg/kg/day) or placebo (saline) for three consecutive days, starting within 24 h of ICH onset. The randomization procedure included adjustment for key prognostic factors including the presenting NIHSS score. An NIHSS score ⩾6 was required for participation, and NIHSS was re-assessed serially on days 2, 3, and 4 (i.e. after each infusion at 24, 48, and 72 h following study drug initiation), as well as on day 7 (or discharge, whichever was earlier) by certified masked investigators. The exact time at which the actual assessments were carried out for each timepoint was also captured. Here, we included all participants comprising the modified-intention-to-treat population of the trial^12^ who had at least one available NIHSS reassessment after presentation.

Further baseline assessments in i-DEF included standardized clinical (demographics/comorbidities) and neuroimaging evaluation. This included central assessment of CT scans by blinded trained raters at the core imaging laboratory to determine hematoma location, intraventricular hemorrhage (IVH) extension, and the volume of hematoma and perihematomal edema. Volumetry was done with an imaging analysis software (Analyze 11.0; AnalyzeDirect, Overland Park, KS, USA) using a validated Hounsfield-unit-threshold-based, semi-automated segmentation approach with manual correction, as described previously.^15^ Follow-up evaluations included functional status on the mRS at 90 and 180 days by certified masked assessors.

### Outcomes

Primary outcome was EMNI_a_, defined as an absolute NIHSS score decrease by ⩾4 points compared to presentation. The NIHSS is a measure of neurological deficits ranging from 0 to 42, with higher scores indicating more severe impairment. We chose NIHSS instead of Glasgow Coma Scale (GCS), another widely used score of clinical severity that focuses on impairment of consciousness, because the former captures neurological impairment in a broader and more detailed manner, and was shown to be a more meaningful assessment tool and better outcome predictor in ICH.^16,17^ We chose a 4-point cutoff, because this definition has long been thought to reflect a clinically important change and is established in ischemic stroke,^18–20^ and has been considered by others for use in ICH.^9^ Since a presenting NIHSS score ⩾6 was required for trial participation, the 4-point-decrement definition was universally applicable in trial participants. Using the NIHSS scores obtained at the prespecified assessment timepoints, we determined the presence or absence of EMNI_a_ as dichotomous outcome on days 2, 3, 4, and 7 by comparing the respective NIHSS scores on days 2, 3, 4, and 7 versus the NIHSS score at presentation.

Secondary outcomes were alternative definitions of EMNI as relative NIHSS score decrement (1) ⩾20% (EMNI_r20_) or (2) ⩾30% (EMNI_r30_) compared to presentation, in accordance with prior research in ischemic stroke showing that relative rather than absolute NIHSS score changes by these thresholds may be more informative.^21,22^ EMNI_r20_ and EMNI_r30_ were again considered as repeated measures on days 2, 3, 4, and 7, as above.

### Statistical analysis

We categorized participants according to a modified-intention-to-treat strategy to deferoxamine versus placebo, in keeping with the main trial.^12^ We present baseline characteristics and outcomes using descriptive statistics, that is, frequencies and percentages for categorical data, and median and interquartile range (IQR) for continuous data. We compared categorical and continuous variables using χ^2^ or Fisher’s exact tests and Mann–Whitney U or t-tests as appropriate, respectively. We performed a longitudinal analysis of EMNI as repeated measure.

#### Primary analysis: association of deferoxamine with EMNI_a_

We used generalized linear mixed-effects models with logit link function and robust standard errors clustered at the participant level. Repeatedly assessed EMNI_a_ during the first 7 days was included as the outcome, participant ID as random intercept, and NIHSS assessment time as continuous variable (exact time for each participant and timepoint, expressed in hours after presentation) as fixed effect. Random slopes were not included due to convergence issues. This approach accounts for the intra-individual correlation of repeated measures and leverages all available data for each participant. It assumes a linear change of logit-EMNI_a_ with time, which was informed by initial exploratory descriptive analysis. To investigate the effect of deferoxamine on EMNI_a_, we fitted the model twice: (1) as a main-effects model, assuming the effect of deferoxamine is constant over time, and (2) as an interaction model to evaluate whether the effect of deferoxamine differs over time. In keeping with the main trial and previous i-DEF subanalyses,^12,23^ we adjusted all models for the prespecified categorical covariates onset-to-treatment time (⩽12 vs >12 h), baseline ICH volume (⩽10 vs >10 mL), and presenting NIHSS score (⩽10 vs >10).

#### Secondary analyses: association of deferoxamine with alternative EMNI definitions

Following the same approach, we used EMNI_r20_ and EMNI_r30_ instead of EMNI_a_ as the outcome in the adjusted linear mixed models described above.

#### Sensitivity analyses

We repeated the primary analysis of EMNI_a_ and the secondary analyses of EMNI_r20_ and EMNI_r30_ (1) alternatively adjusting for presenting NIHSS as continuous variable (instead of the prespecified categories) and (2) maximally adjusting for onset-to-treatment time, baseline ICH volume, and presenting NIHSS, all as continuous variables (after ensuring no gross violation of the linearity assumption), along with IVH presence and ICH location (lobar/thalamic/deep non-thalamic) as categorical ones. This was to account for imbalances of these established outcome predictors between the groups, as in previous i-DEF subanalyses.^13^

#### Additional analyses: EMNI’s determinants and association with long-term outcome

We explored EMNI_a_’s determinants in the entire study population using the same approach as above to investigate the main effects of the following baseline characteristics in a single multivariable generalized linear mixed-effects model adjusted for randomized treatment: presenting NIHSS, age, ICH volume, edema extension distance (EED)^24^ as continuous variables, and sex, race (White/Black/Asian/other), hypertension, diabetes, ICH location (lobar/thalamic/deep non-thalamic), and IVH presence as categorical ones. We repeated these analyses using EMNI_r20_ and EMNI_r30_, instead of EMNI_a_.

We investigated the association of EMNI_a_ at each assessment timepoint with favorable functional outcome at 90 days, defined as mRS score 0–2, in keeping with the main trial.^12,13^ For this, we fitted four separate logistic models for EMNI_a_ on day 2, day 3, day 4, and day 7/(discharge). All models were adjusted for randomized treatment and known outcome predictors including age, presenting NIHSS, ICH volume as continuous variables, and ICH location and IVH presence as categorical ones, as in previous research.^4,6,25^ We repeated these analyses using functional outcome at 180 days. Finally, we repeated all analyses of 90-day and 180-day outcome using EMNI_r20_ and EMNI_r30_ as predictors, instead of EMNI_a_.

For all analyses, we report model-based estimates as odds ratios (ORs) along with 95% confidence intervals (CI) and two-sided p-values. We applied no multiplicity correction given the nature of this post hoc analysis. Analyses were done using STATA v.18 (StataCorp LLC, College Station, TX, USA). We report this study in accordance with the STROBE statement.

#### Handling of missing values

Participants who died and were thus missing NIHSS assessment at any given timepoint were assigned absence of EMNI (using the sample median as assessment time for the purpose of analysis). No other imputations were done in case of missing NIHSS. Baseline data included as model covariates were complete. Given the low missingness in mRS, analysis of EMNI’s association with functional outcome was done on a complete-case basis.

## Results

The entire i-DEF modified-intention-to-treat population comprising 291 participants was available for analysis (median age 61 years, 38.5% female, 147 allocated to placebo, and 144 to deferoxamine; Table 1). Information on presenting NIHSS was complete, with a median score of 13 (IQR 8–18, range 6–33); scores were well balanced between the treatment arms. Besides thalamic ICH and IVH (more common in the placebo arm), the remaining baseline data were reasonably balanced. A detailed comparison of baseline characteristics by treatment allocation has been published previously.^12^

**Table 1.** Baseline characteristics.

	All participants (N = 291)	EMNI_a_ on day 7/(discharge) (N = 275)		
		No EMNI (N = 175)	EMNI (N = 100)	p value
Age, years, median(IQR)	61 (52–70)	62 (53–71)	59 (51–66)	0.17
Female sex, n (%)	112 (38.5%)	61 (34.9%)	46 (46.0%)	0.07
Race, n (%)				0.03
White	181 (62.2%)	120 (68.6%)	51 (51.0%)	
Black	64 (22.0%)	34 (19.4%)	28 (28.0%)	
Asian	37 (12.7%)	16 (9.1%)	17 (17.0%)	
other	9 (3.1%)	5 (2.9%)	4 (4.0%)	
***Medical history***				
Hypertension, n (%)	237 (81.4%)	139 (79.4%)	85 (85.0%)	0.25
Diabetes mellitus, n (%)	75 (25.8%)	40 (22.9%)	30 (30.0%)	0.19
Cardiac disease, n (%)	29 (10.0%)	18 (10.3%)	10 (10.0%)	0.94
Previous ischemic stroke, n (%)	26 (8.9%)	12 (6.9%)	12 (12.0%)	0.15
Previous ICH, n (%)	10 (3.4%)	7 (4.0%)	3 (3.0%)	0.67
***Clinical and radiological data at baseline***				
GCS score, median (IQR)	14 (12–15)	14 (12–15)	14 (12.5–15)	0.26
NIHSS score, median (IQR)	13 (8–18)	13 (9–18)	13 (8–17)	0.92
>10, n (%)	188 (64.6%)	112 (64.0%)	67 (67.0%)	0.62
⩽10, n (%)	103 (35.4%)	63 (36.0%)	33 (33.0%)	
ICH location, n (%)				0.06
lobar	59 (20.3%)	44 (25.1%)	13 (13.0%)	
thalamic	106 (36.4%)	62 (35.4%)	40 (40.0%)	
deep non-thalamic	126 (43.3%)	69 (39.4%)	47 (47.0%)	
ICH volume, mL, median(IQR)	12.9 (6.4–26.0)	17.2 (7.6–34.5)	10.1 (5.5–16.0)	<0.001
>10 mL, n (%)	170 (58.4%)	113 (64.6%)	50 (50.0%)	0.02
⩽10 mL, n (%)	121 (41.6%)	62 (35.4%)	50 (50.0%)	
Intraventricular hemorrhage extension, n (%)	120 (41.2%)	75 (42.9%)	43 (43.0%)	0.98
ICH score, median (IQR)	1 (0–2)	1 (0–2)	1 (0–1)	0.01
EED, mm, median (IQR)	4.4 (3.5–5.3)	4.5 (3.7–5.5)	4.3 (3.4–5.0)	0.06
Treatment allocation, n (%)				0.04
Placebo	147 (50.5%)	96 (54.9%)	42 (42.0%)	
Deferoxamine	144 (49.5%)	79 (45.1%)	58 (58.0%)	
Onset-to-treatment time, h, median(IQR)	18.6 (11–22.8)	19.2 (11.5–23.1)	17.8 (10.6–22.7)	0.40
>12 h, n (%)	201 (69.1%)	122 (69.7%)	70 (70.0%)	0.96
⩽12 h, n (%)	90 (30.9%)	53 (30.3%)	30 (30.0%)	

Early major neurological major improvement on day 7/(discharge was defined as an NIHSS score decrement of an absolute ⩾4 points on day 7/(discharge) compared to presentation; EED, edema extension distance.

Following presentation, all participants had at least one NIHSS reassessment. The median (IQR) time of NIHSS assessments was 26 (24–28), 50 (48–52), 74 (72–77), and 161 (139–169) h after presentation. The median (IQR) NIHSS score was 11 (7–17) on day 2 (11 [7–16] deferoxamine vs 12 [8–18] placebo), and decreased to 10 (6–16) on day 7 (9 [5–15] deferoxamine vs 11 [7–17.5] placebo]. Supplementary Figure 1 shows the detailed trajectory of NIHSS scores. A total of six participants died within 7 days and were therefore classified as “no EMNI” at a total of 7 assessment timepoints following their death. After this imputation, 31 participants with at least one missing NIHSS assessment remained (25 missing one, 6 missing two, and none missing three or more assessments). Among those, NIHSS was missing in 3 participants on day 2, 13 on day 3, 5 on day 4, and 16 on day 7(/discharge).

### Effect of deferoxamine on primary outcome (EMNI_a_)

**Figure 1.**
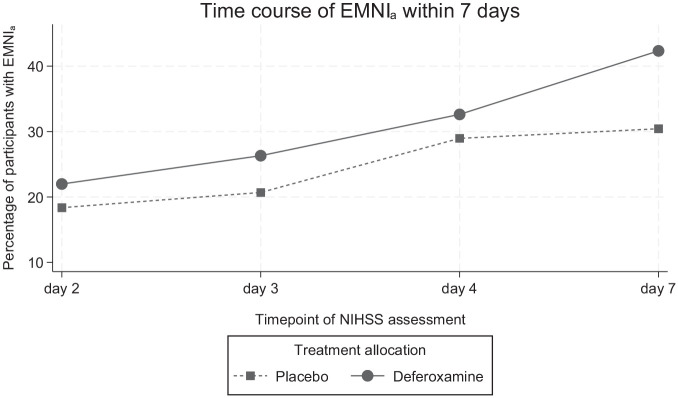
Percentage of participants with early major neurological improvement (EMNI_a_) at the prespecified NIHSS assessment timepoints according to treatment allocation. EMNI_a_ was defined as an NIHSS score decrement of an absolute ⩾4 points between the respective timepoint and presentation.

On day 2, 58/288 participants (20.1%) experienced EMNI_a_ (22% deferoxamine vs 18.4% placebo). This increased to 100/275 (36.4%) on day 7(/discharge) (42.3% deferoxamine vs 30.4% placebo). Figure 1 shows the percentage of participants with EMNI_a_ at consecutive assessment timepoints according to treatment allocation. In the main-effects model of the primary outcome including adjustment for the main trial’s prespecified categorical covariates, deferoxamine showed an average twofold higher odds of EMNI_a_ compared to placebo (OR: 2.30, 95% CI: 1.07–4.95, p = 0.033). The interaction model yielded a weak signal for treatment-by-time interaction, such that the likelihood of EMNI_a_ over time seemed to increase more rapidly with deferoxamine (p_interaction_ = 0.092).

Sensitivity analyses alternatively adjusting the models for continuous presenting NIHSS yielded comparable results (OR: 2.70, 95% CI: 1.24–5.86, p = 0.012 for deferoxamine in the main-effects model, p_interaction_ = 0.089 in the interaction model). Maximally adjusting (for presenting NIHSS, onset-to-treatment time, baseline ICH volume (all continuous), IVH presence, and ICH location) also showed consistent findings (OR 2.11, 95% CI: 0.94–4.74, p = 0.071 for deferoxamine in the main-effects model, p_interaction_ = 0.084 in the interaction model).

### Effect of deferoxamine on secondary outcomes (EMNI_r20_ and EMNI_r30_)

**Figure 2.**
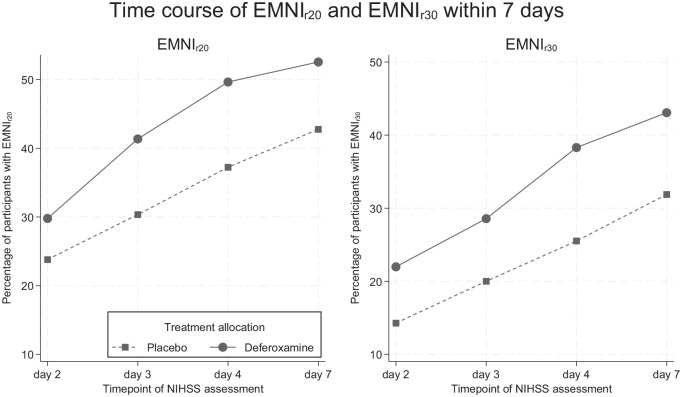
Percentage of participants with early major neurological improvement (left: EMNI_r20_; right: EMNI_r30_) at the prespecified NIHSS assessment timepoints according to treatment allocation. EMNI_r20_ and EMNI_r30_ were defined as an NIHSS score decrement of a relative ⩾20% or ⩾30%, respectively, between the respective timepoint and presentation.

The percentage of participants with EMNI_r20_ and EMNI_r30_ at consecutive assessment timepoints according to treatment allocation is shown in Figure 2. In the main-effects model, adjusted for the trial’s prespecified covariates, deferoxamine showed threefold higher odds of EMNI_r20_ compared to placebo (OR: 2.92, 95% CI: 1.29–6.62, p = 0.010). The interaction model showed no evidence of effect modification by time (p_interaction_ = 0.894). Results were consistent for EMNI_r30_, with the adjusted main-effects model yielding an OR of 4.01 (95% CI: 1.66–9.69, p = 0.002) for deferoxamine’s effect, and the interaction model again showing no evidence of treatment-by-time interaction (p_interaction_ = 0.948). Sensitivity analyses of both secondary outcomes in alternatively and maximally adjusted models showed consistent findings (Supplementary Table 1).

### Determinants of EMNI

**Figure 3.**
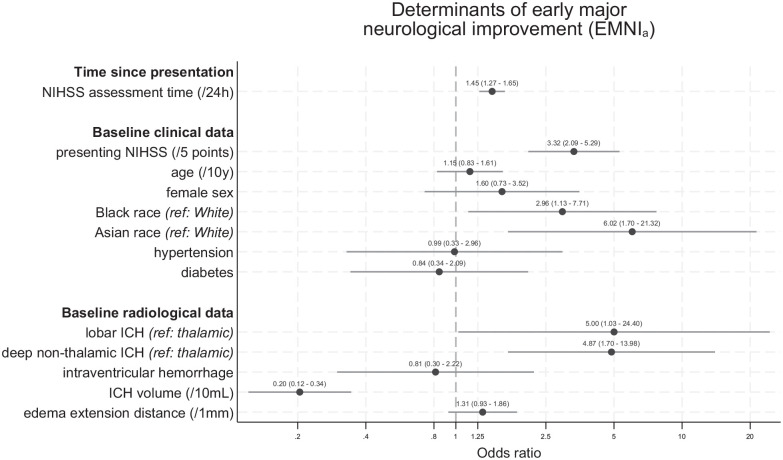
Odds ratio estimates for the association of time and baseline characteristics with EMNI_a_ across all assessment timepoints during the first 7 days following ICH from the linear mixed model. EMNI_a_ was defined at each timepoint as an NIHSS score decrement of an absolute ⩾4 points from presentation.

Baseline characteristics according to EMNI_a_ are given in Table 1. In the multivariable linear mixed model adjusted for randomized treatment, the odds of EMNI_a_ across repeated assessments increased over time (OR: 1.45 per 24 h, 95% CI: 1.27–1.65) and with higher presenting NIHSS (OR: 3.32 per 5 points higher score, 95% CI: 2.09–5.29, both p < 0.001). Both Black (OR: 2.96, 95% CI: 1.13–7.71, p = 0.027) and Asian participants (OR: 6.02, 95% CI: 1.70–21.32, p = 0.005) had higher odds of EMNI_a_ compared to their White counterparts. Larger hematoma volume was associated with lower odds of EMNI_a_ (OR: 0.20 per 10 mL higher volume, 95% CI: 0.12–0.34, p < 0.001), while the odds were higher with both lobar (OR: 5.00, 95% CI: 1.03–24.40, p = 0.046) and deep non-thalamic hematomas (OR: 4.87, 95% CI: 1.70–13.98, p = 0.003) compared to thalamic ones. Figure 3 shows all model-based estimates. Repeated analyses using EMNI_r20_ and EMNI_r30_ yielded consistent results, except for showing no association of presenting NIHSS with EMNI (Supplementary Figure 2).

### Association of EMNI with functional outcome

**Figure 4.**
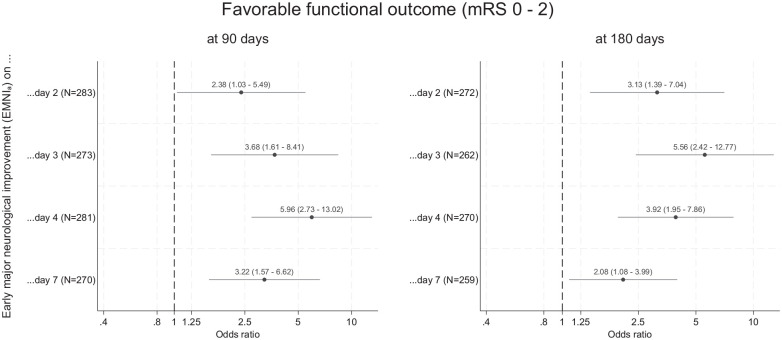
Association of EMNI_a_ at repeated assessments with favorable functional outcome at 90 and 180 days. EMNI_a_ was defined at each assessment timepoint (i.e. day 2, day 3, day 4, or day 7(/discharge)) as an NIHSS score decrement of an absolute ⩾4 points between the respective timepoint and presentation. N indicates the number of participants included in each model.

Data on 90-day mRS were available in 286 of 291 participants (98.3%). Regardless of whether it was assessed on day 2, day 3, day 4, or day 7(/discharge), EMNI_a_ was independently associated with favorable 90-day outcome (mRS 0–2) after adjustment for age, presenting NIHSS, ICH volume, ICH location, IVH, and randomized treatment. The association was strongest for EMNI_a_ on day 4 (OR: 5.96, 95% CI: 2.73–13.02, p < 0.001). We obtained similar results when using 180-day mRS (available in 275/291 participants (94.5%)). Here, the association with favorable 180-day outcome was strongest for EMNI_a_ on day 3 (OR: 5.56, 95% CI: 2.42–12.77, p < 0.001); Figure 4). EMNI_r20_ and EMNI_r30_ showed similar associations with 90-day and 180-day outcome (Supplementary Figures 3 and 4).

## Discussion

This post hoc analysis of the i-DEF trial using serial neurological assessments over 7 days after ICH revealed the following key findings: (1) the likelihood of EMNI was higher with deferoxamine; (2) regardless of treatment, the frequency of EMNI was substantial, grew continuously over time, and was higher in non-White participants, those with higher presenting NIHSS score, smaller and non-thalamic hematomas; (3) EMNI was strongly and independently associated with favorable long-term functional outcome up to 180 days.

Adopting an established definition of EMNI as an absolute NIHSS decrement ⩾4 points, an analytic approach that leverages repeated measures, and prespecified methodological principles of the main trial, we found novel evidence supporting an early treatment effect of deferoxamine on neurological recovery within the first 7 days after ICH in the main-effects model of our primary analysis. Reassuringly, this persisted after additional adjustment for several potential confounders in sensitivity analyses, and was maintained when using alternative—arguably more clinically meaningful^21^—definitions of EMNI as relative (⩾20% or ⩾30%) NIHSS decrements, on which deferoxamine showed even larger effect sizes. Taken together, our findings expand on previous observations from i-DEF that the proportion of participants with early good functional outcome at 7 days was higher with deferoxamine^13^ and bear biological plausibility. Preclinical data indicate that the beneficial effects of deferoxamine in reducing iron accumulation, oxidative damage, and neuroinflammation are evident within this timeframe in ICH animal models.^26–28^ These mechanisms might underlie our findings.

We found no clear evidence for treatment-by-time interaction overall. Some of our interaction analyses yielded weak signals for potential effect modification by time, such that deferoxamine might appear to further accelerate the time-dependent increase in the likelihood of EMNI over the first 7 days following ICH. While power to detect a treatment-by-time interaction may have been low, these findings do not contradict our main results, but rather leave open the possibility that deferoxamine’s effects might become even more pronounced with time. This would be compatible with previous observations from i-DEF that deferoxamine seemed to accelerate the trajectory of longer-term recovery as measured on the mRS.^13^ Finally, these weak signals for interaction might be related to dose-dependent effects, considering that deferoxamine was applied as an early treatment course over the first 3 days after ICH in i-DEF. On the whole, our study offers new data that strengthen the case for deferoxamine as a promising treatment option in ICH and warrant further study. Our findings also highlight the potential of applying longitudinal analyses of repeated outcome measures to clinical trial datasets, as these may improve power to detect treatment effects.^29,30^

We provide a novel comprehensive appraisal of the early course of neurological recovery after ICH, which has been largely unexplored. So far, research focused on neurological worsening, which may occur earlier or later within this timeframe, leads to poor outcome, and may represent a treatment target.^2–4^ However, absence of worsening is not equivalent to improvement, which is worth exploring in its own right. Using a definition of neurological improvement similar to ours but restricted solely between the 24-h and 7-day timepoints, only a single study has recently shown that some degree of subacute improvement is common after ICH.^9^ Expanding on these observations with a granular account of the trajectory of early improvement, we show that a substantial proportion of patients may improve as early as the first day after ICH, and that this proportion will continue to increase up to day 7. This, being in the context of a randomized trial of maximally treated patients and regardless of trial-specific treatments, has several implications: (1) it strengthens the case against early prognostication in ICH,^1^ and (2) it identifies early improvers as a potentially distinct patient subgroup. Future research should determine whether early improvers might be more responsive to treatments with modest therapeutic effects, which may be masked or negated in non-improving, more severely affected patients. In line with this hypothesis, a previous i-DEF subanalysis suggested that deferoxamine’s effects might be confined to patients with medium-sized hematomas, while those with large hematomas did not benefit from treatment.^31^

In that regard, identifying EMNI’s determinants is relevant. We found both smaller hematomas and non-thalamic ones—be it lobar or deep—to carry higher odds of EMNI, which expands on previous data about the adverse prognosis of large hematoma volume^25^ and thalamic location.^32^ We also found that higher presenting NIHSS score was associated with higher odds of EMNI_a_. This is unsurprising, considering that the margin for improvement by that definition (using an absolute score decrement) is larger the higher the baseline score is. Notably, this “reverse ceiling effect” disproportionately favoring EMNI in patients with higher baseline stroke severity was not present when exploring alternative definitions of EMNI as relative NIHSS decrements (EMNI_r20_ and EMNI_r30_), strengthening the case in favor of relative definitions of EMNI.^21,22^ Black and Asian participants showed higher odds of EMNI than their White counterparts in our study. This was after adjustment for established outcome predictors, and might therefore reflect true racial differences in ICH, expanding on previous observations.^25,33^ However, the limited size of these subgroups and substantial uncertainty around the estimates, as reflected in the wide 95% CI, warrant caution, as this may also reflect a chance finding.

Our finding that EMNI—regardless of whether assessed on day 2, 3, 4, or 7 after ICH—is independently associated with favorable long-term functional outcome up to 180 days validates the clinical relevance of EMNI as a phenomenon worthy of study in ICH. Notably, the association with long-term outcomes seemed strongest for EMNI assessed on days 3 and 4 and weaker when assessed on days 2 and 7. This suggests that EMNI may be less clinically significant when assessed too early (i.e. when patients still face a risk of later deterioration) or too late (i.e. when delayed neurological improvement may no longer be sufficient to translate into better functional outcomes). While the reasons for this remain speculative, the timing of clinical improvement has been previously shown to impact long-term outcome after ischemic stroke,^22^ and our data indicate that 48–72 h after presentation might be the optimal time to assess EMNI in ICH. Regardless, these data stress the lasting importance of EMNI and advances recent findings from the INTERACT2 trial, where subacute neurological improvement on day 7 was also associated with functional outcome, albeit up to 90 days.^9^ Of note, NIHSS-based assessment within 1 week was recently proposed as a surrogate endpoint for clinical trial use to reduce trial duration and costs in the field of ischemic stroke.^34^ The benefits of EMNI and shorter time to achieve independence to ICH survivors and their caregivers and healthcare expenses are similarly evident and require further scrutiny.

Our study has several strengths: (1) Although not a prespecified analysis, we applied predetermined methodological aspects of the main trial to explore deferoxamine’s association with outcomes defined using NIHSS scores obtained by certified blinded assessors, thus limiting the risk of spurious findings; (2) several lines of statistical inquiry, different EMNI definitions, and extensive covariate adjustment did not change our main results, underscoring their robustness. We acknowledge the following limitations: (1) the post hoc nature of this analysis and multiple testing limit causal inference and position our findings as hypothesis-generating rather than definitive. (2) The analysis of categorical data as determinants of EMNI is limited by small subgroup sizes, which may reduce statistical power and introduce imprecision and instability into our model-based estimates, warranting caution in their interpretation. (3) Selected using specific eligibility criteria in a phase 2 trial setting, our cohort may be less reflective of the broader ICH patient population and is limited in size. The generalizability/external validity of our findings needs to be assessed in other, larger ICH cohorts.

In conclusion, major neurological improvement starts early and continuously progresses within the first week after ICH in a large proportion of patients and has a strong and lasting association with favorable long-term functional outcome. In this post hoc analysis of the i-DEF trial, treatment with deferoxamine following ICH seemed to enhance EMNI.

## Supplemental Material

sj-pdf-1-wso-10.1177_17474930251348088 – Supplemental material for Early neurological improvement with deferoxamine after intracerebral hemorrhage: A post hoc analysis of the i-DEF trial Supplemental material, sj-pdf-1-wso-10.1177_17474930251348088 for Early neurological improvement with deferoxamine after intracerebral hemorrhage: A post hoc analysis of the i-DEF trial by Alexandros A Polymeris, Vasileios-Arsenios Lioutas, Lydia D Foster, Diego Incontri, Elizabeth C Heistand, Juliette Marchal, Alexa Lazar, Urs Fischer, Stefan T Engelter, David J Seiffge, Sharon D Yeatts and Magdy H Selim in International Journal of Stroke

## References

[1] Greenberg SM Ziai WC Cordonnier C , et al. 2022 guideline for the management of patients with spontaneous intracerebral hemorrhage: a guideline from the American Heart Association/American Stroke Association. Stroke 2022; 53: e282–e361. 10.1161/STR.0000000000000407 35579034

[2] Lord AS Gilmore E Choi HA , et al. Time course and predictors of neurological deterioration after intracerebral hemorrhage. Stroke 2015; 46: 647–652. 25657190 10.1161/STROKEAHA.114.007704 PMC4739782

[3] You S Zheng D Delcourt C , et al. Determinants of early versus delayed neurological deterioration in intracerebral hemorrhage. Stroke 2019; 50: 1409–1414. 31136288 10.1161/STROKEAHA.118.024403

[4] Okazaki S Yamamoto H Foster LD , et al. Late neurological deterioration after acute intracerebral hemorrhage: a post hoc analysis of the ATACH-2 Trial. Cerebrovasc Dis 2020; 49: 26–31. 32045911 10.1159/000506117

[5] Sorimachi T Fujii Y. Early neurological change in patients with spontaneous supratentorial intracerebral hemorrhage. J Clin Neurosci 2010; 17: 1367–1371. 20692165 10.1016/j.jocn.2010.02.024

[6] Yogendrakumar V Smith EE Demchuk AM , et al. Lack of early improvement predicts poor outcome following acute intracerebral hemorrhage. Crit Care Med 2018; 46: e310–e317. 10.1097/CCM.0000000000002962 29303797

[7] Mistry EA Yeatts SD Khatri P , et al. National Institutes of Health Stroke Scale as an outcome in stroke research: value of ANCOVA over analyzing change from baseline. Stroke 2022; 53: e150–e155. 10.1161/STROKEAHA.121.034859 PMC8960347 35012328

[8] Kobeissi H Ghozy S Bilgin C , et al. Early neurological improvement as a predictor of outcomes after endovascular thrombectomy for stroke: a systematic review and meta-analysis. J Neurointerv Surg 2023; 15: 547–551. 35636948 10.1136/neurintsurg-2022-019008

[9] You S Zheng D Chen X , et al. Subacute neurological improvement predicts favorable functional recovery after intracerebral hemorrhage: INTERACT2 study. Stroke 2025; 56: 621–627. 39895502 10.1161/STROKEAHA.124.048847

[10] Bautista W Adelson PD Bicher N , et al. Secondary mechanisms of injury and viable pathophysiological targets in intracerebral hemorrhage. Ther Adv Neurol Disord 2021; 14: 17562864211049208. 10.1177/17562864211049208 PMC8521409 34671423

[11] Wei Y Song X Gao Y , et al. Iron toxicity in intracerebral hemorrhage: physiopathological and therapeutic implications. Brain Res Bull 2022; 178: 144–154. 34838852 10.1016/j.brainresbull.2021.11.014

[12] Selim M Foster LD Moy CS , et al. Deferoxamine mesylate in patients with intracerebral haemorrhage (i-DEF): a multicentre, randomised, placebo-controlled, double-blind phase 2 trial. Lancet Neurol 2019; 18: 428–438. 30898550 10.1016/S1474-4422(19)30069-9 PMC6494117

[13] Foster L Robinson L Yeatts SD , et al. Effect of deferoxamine on trajectory of recovery after intracerebral hemorrhage: a post hoc analysis of the i-DEF trial. Stroke 2022; 53: 2204–2210. 35306827 10.1161/STROKEAHA.121.037298 PMC9246960

[14] Yeatts SD Palesch YY Moy CS , et al. High dose deferoxamine in intracerebral hemorrhage (HI-DEF) trial: rationale, design, and methods. Neurocrit Care 2013; 19: 257–266. 23943316 10.1007/s12028-013-9861-y PMC3932442

[15] Lee KH Lioutas VA Marchina S , et al. The prognostic roles of perihematomal edema and ventricular size in patients with intracerebral hemorrhage. Neurocrit Care 2022; 37: 455–462. 35676589 10.1007/s12028-022-01532-0

[16] Dusenbury W Tsivgoulis G Chang J , et al. Validation of the National Institutes of Health Stroke Scale in intracerebral hemorrhage. Stroke Vasc Interv Neurol 2023; 3: e000834. 10.1161/SVIN.123.000834 PMC12778679 41583670

[17] Kazaryan SA Shkirkova K Saver JL , et al. The National Institutes of Health Stroke Scale is comparable to the ICH score in predicting outcomes in spontaneous acute intracerebral hemorrhage. Front Neurol 2024; 15: 1401793. 39011360 10.3389/fneur.2024.1401793 PMC11246900

[18] Brott TG Haley EC Jr Levy DE , et al. Urgent therapy for stroke. Part I. Pilot study of tissue plasminogen activator administered within 90 minutes. Stroke 1992; 23: 632–640. 1579958 10.1161/01.str.23.5.632

[19] Wityk RJ Pessin MS Kaplan RF , et al. Serial assessment of acute stroke using the NIH Stroke Scale. Stroke 1994; 25: 362–365. 8303746 10.1161/01.str.25.2.362

[20] Irvine HJ Battey TW Ostwaldt AC , et al. Early neurological stability predicts adverse outcome after acute ischemic stroke. Int J Stroke 2016; 11: 882–889. 27334760 10.1177/1747493016654484 PMC5297383

[21] Agarwal S Scher E Lord A , et al. Redefined measure of early neurological improvement shows treatment benefit of alteplase over placebo. Stroke 2020; 51: 1226–1230. 32102629 10.1161/STROKEAHA.119.027476 PMC7101071

[22] Rudilosso S Urra X Amaro S , et al. Timing and relevance of clinical improvement after mechanical thrombectomy in patients with acute ischemic stroke. Stroke 2019; 50: 1467–1472. 31113338 10.1161/STROKEAHA.118.024067

[23] Lioutas V-A Katsanos AH Shoamanesh A , et al. Cognitive outcome after acute spontaneous intracerebral hemorrhage: analysis of the iDEF randomized trial. Cerebrovasc Dis 2024; 54:156–164. 38583421 10.1159/000538415

[24] Parry-Jones AR Wang X Sato S , et al. Edema extension distance: outcome measure for phase ii clinical trials targeting edema after intracerebral hemorrhage. Stroke 2015; 46: e137–e140. 10.1161/STROKEAHA.115.008818 25944323

[25] Woo D Comeau ME Venema SU , et al. Risk factors associated with mortality and neurologic disability after intracerebral hemorrhage in a racially and ethnically diverse cohort. JAMA Network Open 2022; 5: e221103. 10.1001/jamanetworkopen.2022.1103 PMC8924717 35289861

[26] Nakamura T Keep RF Hua Y , et al. Deferoxamine-induced attenuation of brain edema and neurological deficits in a rat model of intracerebral hemorrhage. J Neurosurg 2004; 100: 672–678. 15070122 10.3171/jns.2004.100.4.0672

[27] Gu Y Hua Y Keep RF , et al. Deferoxamine reduces intracerebral hematoma-induced iron accumulation and neuronal death in piglets. Stroke 2009; 40: 2241–2243. 19372448 10.1161/STROKEAHA.108.539536 PMC2693321

[28] Xie Q Gu Y Hua Y , et al. Deferoxamine attenuates white matter injury in a piglet intracerebral hemorrhage model. Stroke 2014; 45: 290–292. 24172580 10.1161/STROKEAHA.113.003033 PMC3886635

[29] Feng W Vasquez G Suri MFK , et al. Repeated-measures analysis of the national institute of neurological disorders and stroke rt-PA stroke trial. J Stroke Cerebrovasc Dis 2011; 20: 241–246. 20621509 10.1016/j.jstrokecerebrovasdis.2010.01.003

[30] Fan L Yeatts SD Foster LD , et al. Endovascular therapy demonstrates benefit over intravenous recombinant tissue plasminogen activator based on repeatedly measured National Institutes of Health Stroke Scale. Interv Neurol 2017; 6: 25–30. 28611830 10.1159/000452137 PMC5465731

[31] Wei C Wang J Foster LD , et al. Effect of deferoxamine on outcome according to baseline hematoma volume: a post hoc analysis of the i-DEF trial. Stroke 2022; 53: 1149–1156. 34789008 10.1161/STROKEAHA.121.035421 PMC8960321

[32] Delcourt C Sato S Zhang S , et al. Intracerebral hemorrhage location and outcome among INTERACT2 participants. Neurology 2017; 88: 1408–1414. 28235817 10.1212/WNL.0000000000003771 PMC5386433

[33] Krishnan K Beishon L Berge E , et al. Relationship between race and outcome in Asian, Black, and Caucasian patients with spontaneous intracerebral hemorrhage: data from the Virtual International Stroke Trials Archive and Efficacy of Nitric Oxide in Stroke trial. Int J Stroke 2018; 13: 362–373. 29165060 10.1177/1747493017744463

[34] Chalos V van der Ende NAM Lingsma HF , et al. National Institutes of Health Stroke Scale: an alternative primary outcome measure for trials of acute treatment for ischemic stroke. Stroke 2020; 51: 282–290. 31795895 10.1161/STROKEAHA.119.026791 PMC6924951

