# Supplementary material for: Early neurological improvement with deferoxamine after intracerebral hemorrhage: A post hoc analysis of the i-DEF trial
Source: Int J Stroke. 2025 May 26;20(10):1235–45. doi: 10.1177/17474930251348088 (PMC12664922; doi:10.1177/17474930251348088)
Supplement: sj-pdf-1-wso-10.1177_17474930251348088 – Supplemental material for Early neurological improvement with deferoxamine after intracerebral hemorrhage: A post hoc analysis of the i-DEF trial [file sj-pdf-1-wso-10.1177_17474930251348088.pdf]

Supplementary Material

Early neurological improvement with deferoxamine after intracerebral hemorrhage: a post hoc analysis of the i-DEF trial

Polymeris A, Lioutas VA, Foster LD, Incontri D, Heistand EC, Marchal J, Lazar A, Fischer U, Engelter ST, Seiffge DJ, Yeatts SD, Selim MH for the i-DEF Investigators

Supplementary Table 1. Sensitivity analyses of secondary outcomes using additional adjustments

**Secondary outcome**	**Model adjustment**	**Effect of deferoxamine** (main effects model)	**Treatment-by-time interaction** (interaction model)
$EMNI_{r20}$	alternatively adjusted	OR 2.80 (95%-CI 1.23-6.34) p=0.014*	$p_{interaction}$=0.887‡
	maximally adjusted	OR 2.03 (95%-CI 0.88-4.67) p=0.095†	$p_{interaction}$=0.848‡
$EMNI_{r30}$	alternatively adjusted	OR 3.76 (95%-CI 1.56-9.07) p=0.003*	$p_{interaction}$=0.940‡
	maximally adjusted	OR 2.89 (95%-CI 1.12-7.50) p=0.029†	$p_{interaction}$=0.875‡

* includes participant ID as random intercept and NIHSS assessment time, treatment allocation (deferoxamine vs placebo), onset-to-treatment time (categorized to ≤12 vs >12 h), baseline ICH volume (categorized to ≤10 vs >10 mL), and presenting NIHSS (as continuous variable) as fixed effects

† includes participant ID as random intercept and NIHSS assessment time, treatment allocation (deferoxamine vs placebo), onset-to-treatment time, baseline ICH volume, and presenting NIHSS (all as continuous variable), as well as IVH presence and ICH location (categorized to lobar, thalamic, or deep non-thalamic) as fixed effects

‡ adjustment as in the respective main effects model, but with an additional interaction term NIHSS assessment time x treatment allocation

Supplementary Figure 1. Trajectory of NIHSS score over the first 7 days following ICH according to treatment allocation.

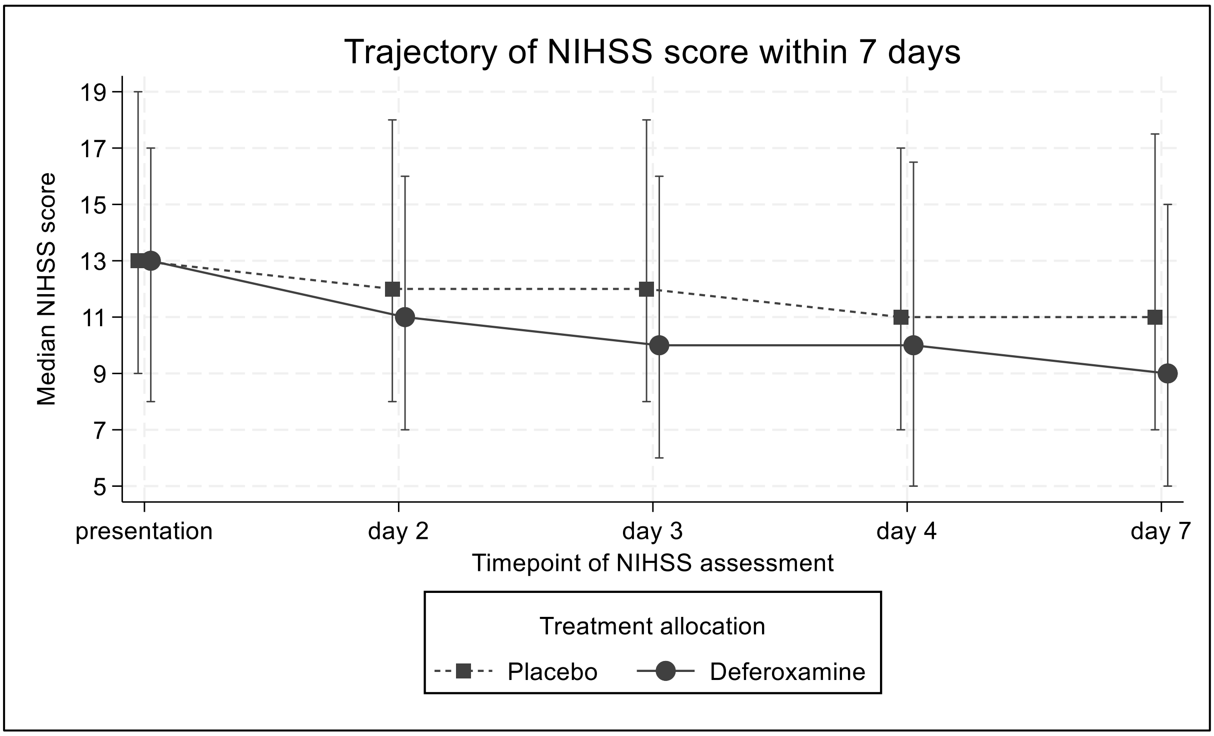

Points represent median NIHSS scores; vertical bars represent the interquartile range.

Supplementary Figure 2. Odds ratio estimates for the association of time and baseline characteristics with EMNI$_{r20}$ and EMNI$_{r30}$ across all assessment timepoints during the first 7 days following ICH from the linear mixed model. EMNI$_{r20}$ and EMNI$_{r30}$ were defined at each timepoint as an NIHSS score decrement of a relative ≥20% or ≥30% from presentation, respectively.

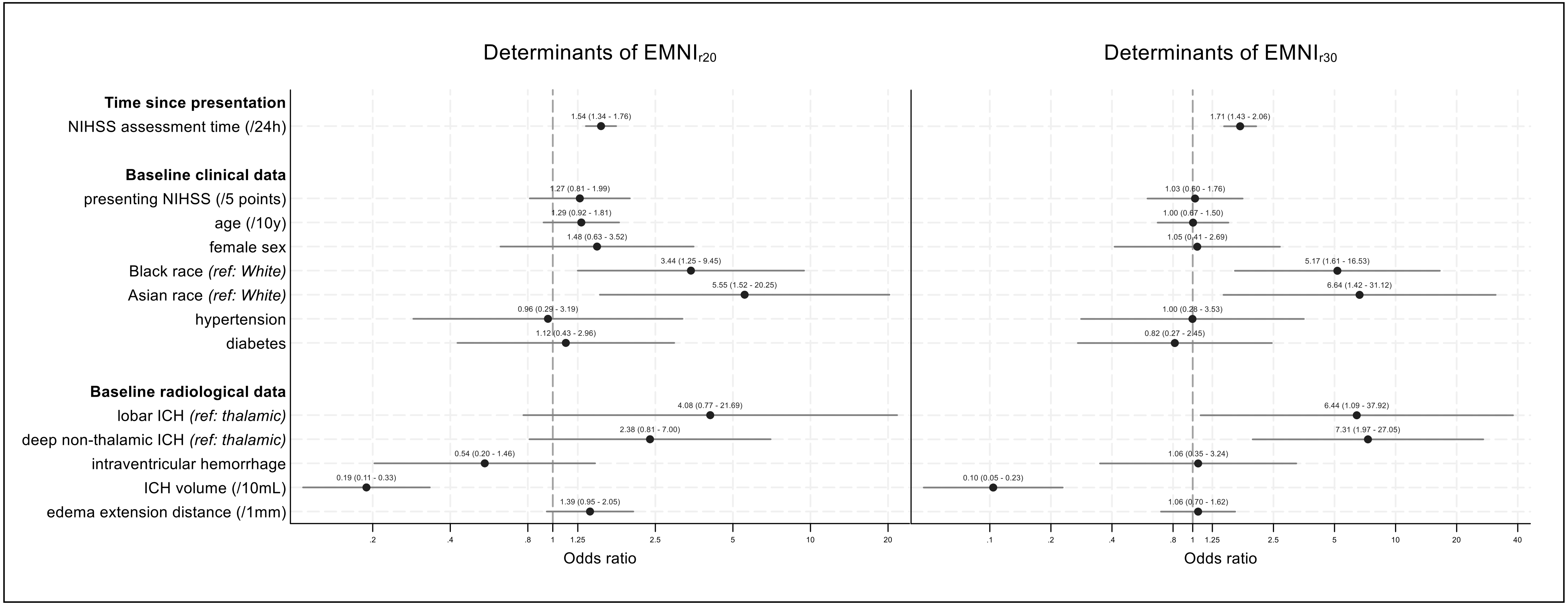

Supplementary Figure 3. Association of $EMNI_{r20}$ at repeated assessments with favorable functional outcome at 90 and 180 days. $EMNI_{r20}$ was defined at each assessment timepoint [i.e., day-2, day-3, day-4, or day-7(/discharge)] as an NIHSS score decrement of a relative ≥20% between the respective timepoint and presentation.

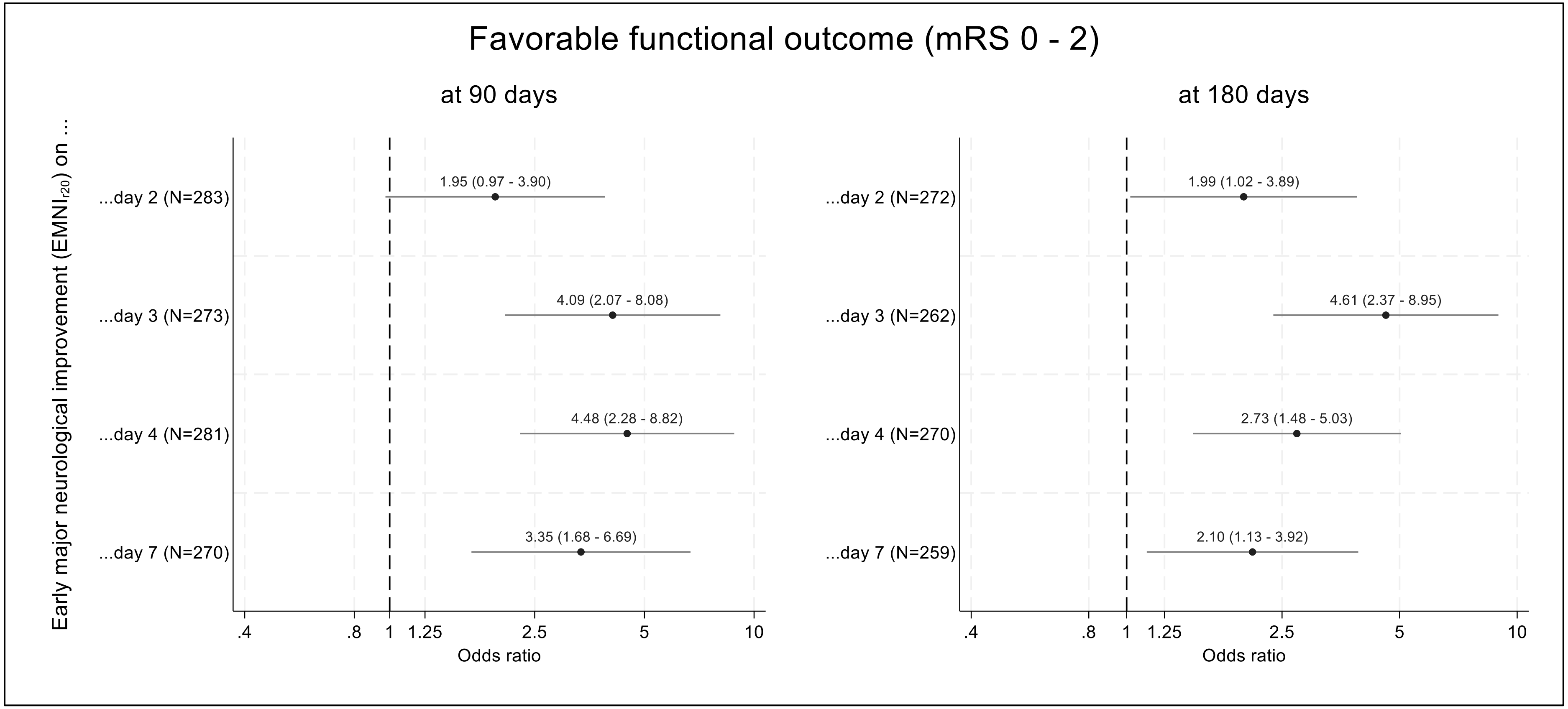

N indicates the number of participants included in each model.

Supplementary Figure 4. Association of $EMNI_{r30}$ at repeated assessments with favorable functional outcome at 90 and 180 days. $EMNI_{r30}$ was defined at each assessment timepoint [i.e., day-2, day-3, day-4, or day-7(/discharge)] as an NIHSS score decrement of a relative ≥30% between the respective timepoint and presentation.

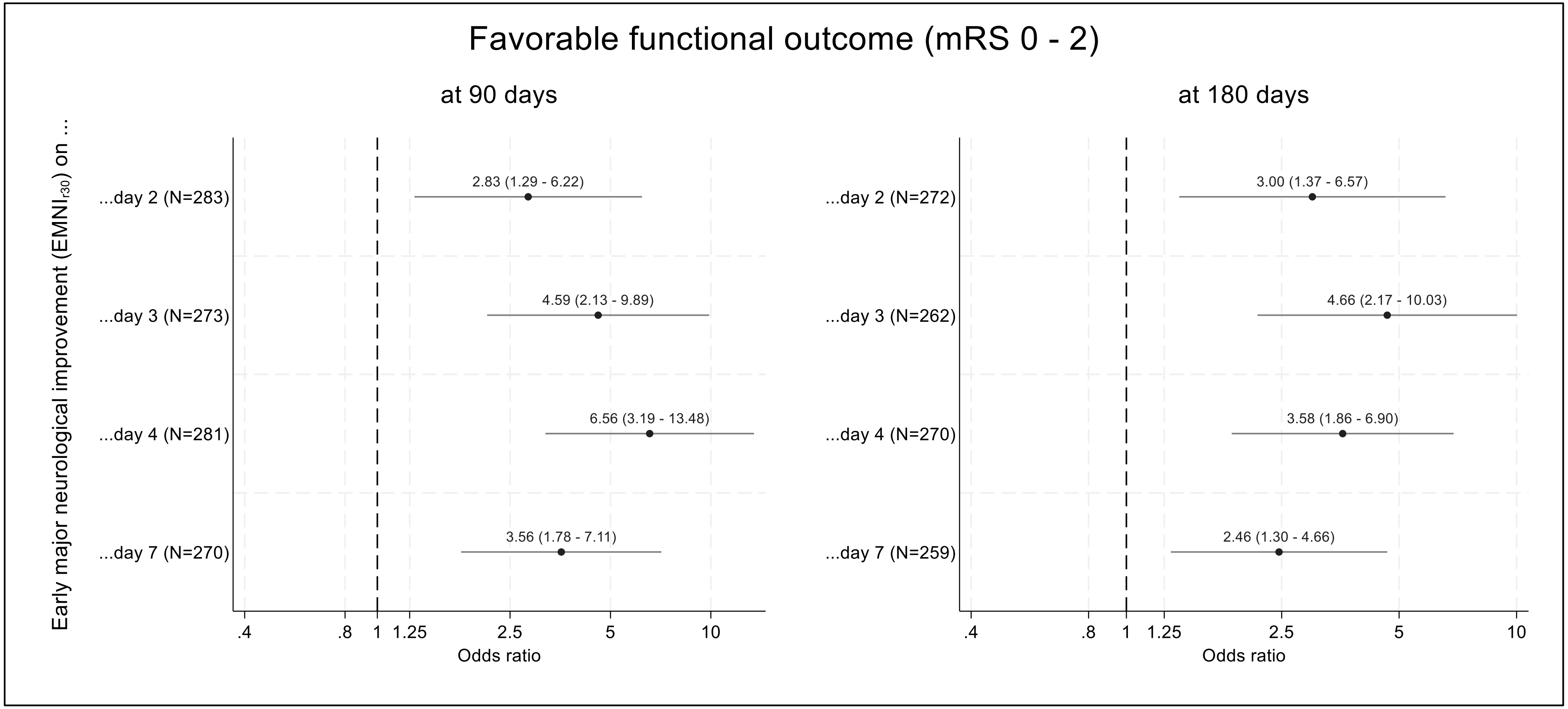

N indicates the number of participants included in each model.
